# PARP7 and Mono-ADP-Ribosylation Negatively Regulate Estrogen Receptor α Signaling in Human Breast Cancer Cells

**DOI:** 10.3390/cells10030623

**Published:** 2021-03-11

**Authors:** Marit Rasmussen, Susanna Tan, Venkata S. Somisetty, David Hutin, Ninni Elise Olafsen, Anders Moen, Jan H. Anonsen, Denis M. Grant, Jason Matthews

**Affiliations:** 1 Department of Nutrition, Institute of Basic Medical Sciences, Faculty of Medicine, University of Oslo, Sognsvannsveien 9, 0372 Oslo, Norway; marit.rasmussen@medisin.uio.no (M.R.); satheeshkumarsv@gmail.com (V.S.S.); n.e.olafsen@studmed.uio.no (N.E.O.); 2 Department of Pharmacology and Toxicology, University of Toronto, 1 King’s College Circle, Toronto, ON M5S 1A8, Canada; stan@bccrc.ca (S.T.); dhutin1@gmail.com (D.H.); denis.grant@utoronto.ca (D.M.G.); 3 Department of Biosciences, Faculty of Mathematics and Natural Sciences, University of Oslo, Blindernveien 31, 0371 Oslo, Norway; anders@hava.no (A.M.); j.h.anonsen@ibv.uio.no (J.H.A.)

**Keywords:** PARP7, ARTD14, TIPARP, mono-ADP-ribosylation, estrogen receptor α, poly ADP-ribose polymerase, breast cancer

## Abstract

ADP-ribosylation is a post-translational protein modification catalyzed by a family of proteins known as poly-ADP-ribose polymerases. PARP7 (TIPARP; ARTD14) is a mono-ADP-ribosyltransferase involved in several cellular processes, including responses to hypoxia, innate immunity and regulation of nuclear receptors. Since previous studies suggested that PARP7 was regulated by 17β-estradiol, we investigated whether PARP7 regulates estrogen receptor α signaling. We confirmed the 17β-estradiol-dependent increases of PARP7 mRNA and protein levels in MCF-7 cells, and observed recruitment of estrogen receptor α to the promoter of PARP7. Overexpression of PARP7 decreased ligand-dependent estrogen receptor α signaling, while treatment of PARP7 knockout MCF-7 cells with 17β-estradiol resulted in increased expression of and recruitment to estrogen receptor α target genes, in addition to increased proliferation. Co-immunoprecipitation assays revealed that PARP7 mono-ADP-ribosylated estrogen receptor α, and mass spectrometry mapped the modified peptides to the receptor’s ligand-independent transactivation domain. Co-immunoprecipitation with truncated estrogen receptor α variants identified that the hinge region of the receptor is required for PARP7-dependent mono-ADP-ribosylation. These results imply that PARP7-mediated mono-ADP-ribosylation may play an important role in estrogen receptor positive breast cancer.

## 1. Introduction

The poly-ADP-ribose polymerase (PARP) family consists of 17 enzymes that use nicotinamide adenine dinucleotide (NAD^+^) as a substrate to transfer ADP-ribose onto themselves and target proteins [1,2]. This activity depends on the conserved histidine-tyrosine-glutamate (HYE) catalytic triad motif, although the glutamate residue is absent in 11 of the protein family members, suggesting that they differ in their catalytic activity [3,4]. The majority of PARPs catalyze the transfer of one ADP-ribose monomer, a process known as mono-ADP-ribosylation [2]. Several bacterial toxins exert their pathogenic mechanisms by acting as mono-ADP-ribosyltransferases (mARTs), including diphtheria [5], giving rise to the alternative nomenclature diphtheria-toxin-like ADP-ribosyltransferases (ARTDs). Generally, ADP-ribosylation can alter a target protein’s activity, stability and turnover, and the modification may affect cellular stress responses, DNA repair, immunity, transcription and metabolism [6,7,8]. ADP-ribosylation is removed by enzymes such as poly-ADP-ribose glycohydrolases (PARGs), ADP-ribosyl hydrolases (ARHs) and macro domain containing proteins, making the modification reversible [8,9,10].

PARP7 (TIPARP; ARTD14), is a mono-ADP-ribosyltransferase that is a critical regulator of innate immunity, transcription factor activity, and cellular stress responses [11,12]. PARP7 is expressed in most human tissues, and has an N-terminal nuclear localization signal (NLS), followed by a cysteine-cysteine-cysteine-histidine (CCCH)-type zinc finger domain which can bind RNA, a tryptophan-tryptophan-glutamate (WWE) domain which can bind ADP-ribose and mediate protein-protein interactions, and a conserved PARP domain responsible for its enzymatic activity [3,13,14,15,16]. Expression of PARP7 is regulated by the aryl hydrocarbon receptor (AHR), and PARP7 acts as a repressor of AHR activity via mono-ADP-ribosylation [17]. PARP7 is also regulated by liver X receptors (LXRs) [18], hypoxia-inducible factor 1 (HIF-1α) [19], and the type I interferon (IFN-I) response during viral infection [20]. Recently, a potent and selective small molecule inhibitor of PARP7, RBN-2397, was reported to enhance IFN-I signaling and cause lung cancer regression in xenograft models [21]. 

CRISPR-Cas9 screens have identified PARP7 as a potential therapeutic target for several human cancers [22]. Compared with healthy tissue, PARP7 expression is reduced in a range of cancers, including breast cancer where higher PARP7 levels have been associated with a better outcome. PARP7 is expressed at higher levels in estrogen receptor (ER) and progesterone receptor (PR) positive breast tumors compared with ER and PR negative breast tumors [22]. Moreover, patients with advanced stages of breast cancer have lower expression levels of PARP7 [22]. 

Estrogen receptor α (ERα) is the dominant regulator of estrogen action in breast tissue maintenance and mammary gland development [23], and the principal therapeutic target for breast cancer treatment [24]. ERα contains several structurally conserved domains that are important for its functions. The A/B domains contain the activation function 1 (AF-1) region that facilitates ligand-independent activation. The DNA binding domain (DBD) is located in the C domain and is involved in binding to estrogen response elements (EREs) found in the regulatory regions of ER target genes. The D domain, known as the hinge region, acts as a flexible linker important for correct conformational changes, and contains a putative NLS. The E domain contains the ligand-dependent AF-2 region and the ligand-binding domain (LBD) [25,26]. Recent studies have suggested that 17β-estradiol (E2) induces expression of PARP7, and that PARP7 promotes the proteolytic degradation of ERα [19]; however, the underlying mechanisms are not well understood. In this study, we sought to investigate whether PARP7 regulates ERα by mono-ADP-ribosylation. Our findings show that ERα regulates PARP7 expression, and that PARP7 acts as a negative regulator of ERα activity via mono-ADP-ribosylation in human breast cancer cells.

## 2. Materials and Methods

### 2.1. Chemicals

The chemicals dimethyl sulfoxide (DMSO), 17β-estradiol (E2), and 4-hydroxytamoxifen (4-OHT) were purchased from Sigma-Aldrich (St. Louis, MO, USA). RBN-2397 was purchased from MedChemExpress (Monmouth Junction, NJ, USA). All other chemicals were purchased from Sigma-Aldrich unless stated otherwise. 

### 2.2. Plasmids

The plasmids pGEX-PARP7, pEGFP-PARP7, pEGFP-PARP7^H532A^, pSG5-ERα, pcDNA3.1-PARP7 and pcDNA3.1-PARP7^H532A^ have been described elsewhere [13,17,27]. pCMV-FLAG-ERα, pCMV-3xFLAG-ERα ABC, pCMV-3xFLAG-ERα ABCD, and pCMV-3xFLAG-ERα CDEF were made by PCR based cloning using the following PCR primers: ERα forward 5′-CAAAGAATTCATGACCATGACCCTCCACACCA-3′: ERα reverse 5′-CAAACTCGAGTCAGACCGTGGCAGGGAAACC-3′: ERα A forward 5′-CAAAGAA TTCCATGACCATGACCCTCCACACCA-3′: ERα C forward 5′-CAAAGAATTCCGAGACTCGCTACTGTGCAGTGT-3′: ERα C reverse 5′-CAAAGGATCCTCACATCATTCC CACTTCGTAGCATTTGC-3′: ERα D reverse 5′-CAAAGGATCCTCAAGAGCGTTTGAT CATGAGCGGGCT-3′: ERα F reverse 5′-CAAAGGATCCTCAGACCGTGGCAGGG AAACC-3′. Restriction enzyme recognition sites are underlined in the primers. The amplified sequences were digested with *Eco*RI and *Xho*I, or *Eco*RI and *Bam*HI, and cloned into either pCMV-FLAG or pCMV-3xFLAG, respectively. 

### 2.3. Cell Culturing

The MCF-7, MCF-7 PARP7-HA, COS-1, MDA-MB-231, HuH-7 and mouse embryonic fibroblast (MEFs) cell lines were used in these studies. MCF-7 cells are ERα positive luminal A subtype breast cancer cells routinely used to study ERα signaling. The generation of the doxycycline (DOX)-inducible PARP7-hemagglutinin (HA) overexpressing MCF-7 cell line (MCF-7 PARP7-HA) has been previously described [13]. HuH-7 human hepatoma cells were used because they are ERα negative and easily transfected at high efficiency. MDA-MB-231 cells are triple negative breast cancer cells that are ERα negative. COS-1 cells are African green monkey kidney fibroblast-like cells that are transfected at high efficiency, and we were able to overexpress PARP7 at higher levels in these cells compared with MCF-7 or HuH-7 cells. Isolation and immortalization of *Parp7^+/+^* and *Parp7^−/−^* MEFs have been described elsewhere [17]. Generation of the *Parp7^H532A^* mice by CRISPR-Cas9 gene editing is described elsewhere (Hutin, D. Long, A., Sugamori, K, Shao, P., Hagen, K.A., Grimaldi, G., Grant, D.M. and Matthews, Jason, unpublished data). *Parp7^H532A^* (*Tiparp^H532A^*) mice were designed and created by Cyagen (Santa Clara, CA, USA). Briefly, a gRNA sequence was designed to target the amino acid residue H532 located in exon 6 of murine *Parp7*. An oligo donor with targeting sequence, flanked by 60 bp homologous sequence containing the H532A (CAT to GCC) mutation was introduced into exon 6 by homology-directed repair. Once the mutation was confirmed, the mouse colony was expanded and maintained by breeding *Parp7*^+/H532A^ heterozygous mice. The generation of *Parp7^H532A^* MEFs isolated from these mice was done as previously described [17]. 

All cell lines were cultured in DMEM (1.0 g/L glucose), supplemented with 10% *v*/*v* fetal bovine serum (FBS), 1% *v*/*v* L-glutamine and 1% *v*/*v* penicillin-streptomycin (P/S). Cells were maintained at 37 °C, with 100% humidity and 5% CO_2_, and subcultured when 80% confluence was reached. For experiments involving estrogenic compounds, cells were starved in phenol red-free DMEM (1.0 g/L glucose), supplemented with 5% *v*/*v* dextran-coated charcoal (DCC)-stripped FBS, 1% *v*/*v* L-glutamine and 1% *v*/*v* P/S for at least 48 h before treatment with ligand.

### 2.4. Real Time qPCR (RT-qPCR)

RNA was isolated using Aurum™ Total RNA isolation kit (BioRad, Hercules, CA, USA), and was subsequently used to synthesize cDNA according to the manufacturer’s protocol (Applied Biosystems, Foster City, CA, USA). Synthesized cDNA was diluted 1:3 in dH_2_O. Each reaction consisted of 0.1 µL forward primer, 0.1 µL reverse primer, 5 µL 2X KAPA SYBR^®^ FAST (Kapa Biosciences, Wilmington, MA, USA), 1 µL of the diluted cDNA and dH_2_O to a total volume of 10 µL. Reactions were set up in three technical replicates, and loaded on 96-well PCR plates. All target transcripts were normalized to the housekeeping gene TATA-binding protein (TBP), and further analyzed using the comparative cycle threshold (CT) (ΔΔCT) method. Target transcript expression levels are shown as fold change in comparison to the DMSO-treated wildtype samples. The primers used were TBP: forward 5′-TTGTACCGCAGCTGCAAAAT-3′ and reverse 5′-TATATTCG GCGTTTCGGGCA-3′, PARP7: forward 5′-GGCAGATTTGAATGCCATGA-3′ and reverse 5′-TGGACAGCCTTCGTAGTTGGT-3′, Growth regulating estrogen receptor binding 1 (GREB1): forward 5′-CAAAGAATAACCTGTTGGCCCTGC-3′ and reverse 5′-GACATG CCTGCGCTCTCATACTTA-3′, Trefoil factor 1 (TFF1): forward 5′-CATCGACGTCCCT CCAGAAGAG-3′ and reverse 5′-CTCTGGGACTAATCACCGTGCTG-3′, and Cytochrome P450 family 1 subfamily A member 1 (CYP1A1): forward 5′-TGGTCTCCCTTCTC TACACTCTTGT-3′ and reverse 5′-ATTTTCCCTATTACATTAAATCAATGGTTCT-3′. 

### 2.5. Chromatin Immunoprecipitation

Cells were plated in 10 cm dishes at a density of 2 × 10^5^ cells per mL. For studies using MCF-7 cells, cells were exposed to test ligands 48 h after serum starvation. For assays with overexpressed ERα and PARP7, HuH-7 cells were transfected with a total of 2.5 µg DNA consisting of 300 ng of pSG5-ERα and either 2.2 µg of pEGFP-PARP7, pEGFP-PARP7^H532A^ or 7.5 ng of pEGFP and 2.2 µg of pcDNA3.1 using Lipofectamine 3000 (Thermo Fisher Scientific, Waltham, MA, USA). Cells were treated with DMSO or E2 for one hour, and formaldehyde was added to a final concentration of 1% and cells were left on a shaker for 10 min. Glycine was added to a final concentration of 0.125 M, and plates were left on the shaker for 5 min. Preparation of the cell extract and ChIP assay was performed essentially as we have previously described using a negative control (no antibody; MCF-7 only) or rabbit IgG (Sigma-Aldrich; HuH-7 only), 3 µg of anti-GFP (Thermo Fisher Scientific; 3E6) or 3 µg of anti-ERα (Santa Cruz Biotechnology, Dallas, TX, USA; HC-20) per immunoprecipitation [28]. One µL from each sample and the input samples were analyzed by RT-qPCR. The primers used were *PARP7*: forward 5′-TTTGCTTCCTCACAGGGTGT-3′ and reverse 5′-AGGGTCACTTTGTTCCGAGA-3′, *GREB1*: forward 5′-CCAGGCTGCCAGCT GACT-3′ and reverse 5′-CAAAGGGTCAGGAGAAGAACACA-3′, and *TFF1*: forward 5′-CCGGCCATCTCTCACTATGAA-3′ and reverse 5′-CCTCCCGCCAGGGTAAATAC-3′.

### 2.6. Generation of Anti-PARP7 Antibody

6xHistidine-tagged murine Parp7 (mParp7) 1–320 was expressed in *E. coli* (BL-21) using pET vector and purified in 6 M guanidine with HisPur Cobalt Resin (Pierce, Rockford, IL, USA) and eluted with imidazole. Recombinant mParp7 was dialyzed for one hour against 20 mM acetic acid. Eight-week-old female BALB/C mice were immunized three times at 2-week intervals with 50 µg of protein in RIBI adjuvant (Millipore Sigma, Burlington, MA, USA) followed by 2 injections with 20 µg in RIBI adjuvant. Immunization was assessed by ELISA against 6xHistidine-tagged mParp7 1–320 and mice were given a booster dose (10 µg protein in PBS) 3 days before the fusion of spleen cells with SP2/O myeloma cells with PEG 1500 (Roche, Basel, Switzerland). Hybridomas producing specific antibodies recognizing mPARP7 were screened by ELISA on plates coated with the recombinant protein and cloned by limiting dilutions. The selected clone which recognizes mParp7 by western blot was purified with HiTrap Protein G HP (Millipore, Oakville, Canada). 

### 2.7. Western Blotting

MCF-7 cells were seeded in six-well plates at a density of 2 × 10^5^ cells per well. Forty-eight hours after serum starvation, cells were treated with test ligands for 4 or 24 h. Cells were lysed in RIPA buffer supplemented with 1X PIC and sonicated at a low intensity for 2 × 30 s on/off. The protein concentration of the clarified lysate was determined using the BCA assay according to the manufacturer’s protocol (Thermo Fisher Scientific). 40 µg of protein was separated by SDS-PAGE and transferred to polyvinylidene fluoride (PVDF) membranes. COS-1 cells were transfected with 1 µg of pEGFP-PARP7, pEGFP-PARP7 33–657, pEGFP-PARP7 53–657, pEGFP-PARP7 103–657, pEGFP-PARP7 200–657, pEGFP-PARP7 235–657, or pEGFP-PARP7 1–234. MEFs were seeded in six-well plates at a density of 1.0 × 10^5^ cells per mL. MCF-7 PARP7-HA cells were seeded in six-well plates at a density of 1.2 × 10^5^ cells per mL. After 24 h, these cells were incubated with and without 1.5 μg/mL DOX. The following day, cells were harvested and lysed in lysis buffer (200 mM NaCl, 20 mM Hepes, 1% Nonidet P-40), 20 µg of protein was separated by SDS-PAGE and transferred to PVDF membranes. Membranes were incubated with lab-generated anti-PARP7, anti-PARP7 (Abcam, Cambridge, UK; ab84664; lot# GR3304056-5), anti-ERα (MCF-7 cells only; Santa Cruz Biotechnology; HC-20), anti-GFP (COS-1 only; Clontech Laboratories, Mountain View, CA, USA; JL-8, anti-HA (MCF-7 PARP7-HA cells only; BioLegend, San Diego, CA, USA; 16B12) and anti-β-actin (Sigma-Aldrich; AC-74). After incubation with appropriate secondary antibody, bands were visualized with SuperSignal^TM^ West Dura Extended Duration Substrate or SuperSignal^TM^ West Femto Maximum Sensitivity Substrate (Thermo Fisher Scientific).

### 2.8. Reporter Gene Assay

HuH-7 cells were transfected with 400 ng ERE-TK-Luc, 2 ng pSG5-ERα, 100 ng β-galactosidase and either 50, 100, 200 or 400 ng of pcDNA3.1-PARP7, or with 200 ng of the catalytically inactive pcDNA3.1-PARP7^H532A^. MCF-7 cells were transfected with 400 ng ERE-TK-Luc, 2 ng pSG5-ERα, 100 ng β-galactosidase and either 200 ng of pcDNA3.1-PARP7, or with 200 ng of pcDNA3.1-PARP7^H532A^. Both cell lines were transfected using Lipofectamine 2000 (Thermo Fisher Scientific) and treated with test ligands 24 h later. Reporter gene assays were carried out the following morning, as described previously [29].

### 2.9. Generation of Knockout Cells

The generation of MCF-7 AHR^KO^ cells has been described elsewhere [30]. MCF-7 PARP7^KO^ cells were generated using CRISPR-Cas9. The guide RNAs (gRNAs) used to target PARP7 were designed to target exon 2 of the *PARP7* gene (NC_000069.7) using the Broad Institute’s gRNA design tool (https://portals.broadinstitute.org/gpp/public/analysis-tools/sgrna-design, accessed on 17 March 2017. The following guide oligos were designed to express the sgRNA: forward primer 5′-GGAGGCTGCACTACACAGTC-3′ and reverse primer 5′-GACTGTGTAGTGCAGCCTCC-3′. The gRNA was cloned into the pSpCas9(BB)-2A-Puro (PX459) plasmid (Addgene, Watertown, MA, USA; plasmid #62988), containing *Streptococcus pyrogenes* (Sp) Cas9 and puromycin genes. The efficiency of the gRNA at targeting *PARP7* was assessed with T7 endonuclease assays. The PX459 PARP7 containing gRNA plasmid was transfected into MCF-7 cells, using Lipofectamine 2000 (Thermo Fisher Scientific). Three days after transfection, the cells were exposed to 1 µg/mL puromycin for 4 days. The puromycin was then removed and the transfected cells were cultured for an additional 7 days before dilution in order to isolate MCF-7 PARP7^KO^ clones. To confirm the knockout, genomic DNA was isolated from MCF-7 PARP7^KO^ cells and the regions surrounding the gRNA binding site in *PARP7* was PCR amplified using the primers: forward 5′-CATCTTCCTTCCTTTCCTCGTA-3′ and reverse 5′-CTAAAAACCCCATCAAGTGAGC-3. The amplicon was cloned into pGEM-T Easy vector (Promega, Madison, WI, USA) and 45 individually cloned genomic DNA amplicons were sequenced to confirm knockout. Using this approach, we identified one clone containing insertions and deletions resulting in frameshift mutations.

### 2.10. Cell Proliferation Assay

MCF-7 cells were plated in phenol red-free DMEM with DCC-stripped FBS in 96-well plates at a density of 8 × 10^3^ cells per well. The following day, cells were exposed to test ligands. Proliferation was measured on day 1 (baseline) and day 4 with CellTiterGlo^®^ Luminescent Cell Viability Assay according to the manufacturer’s protocol (Promega). Relative proliferation was determined by normalizing the luminescence output from day 4 to day 1 and presented as % of DMSO-treated cells. 

### 2.11. Co-Immunoprecipitation

COS-1 cells were seeded in six-well plates at a density of 2 × 10^5^ cells per well. The following day, cells were transfected with 0.5 µg of pCMV-FLAG-ERα, and either 1 µg pEGFP-PARP7, 0.8 µg pEGFP-PARP7^H532A^ or 0.1 µg of pEGFP using Lipofectamine 2000. Various amounts of pcDNA3.1 was used to reach a total amount of 1.5 µg DNA. Cells were treated with E2 for 24 h. For the truncated ERα variants, cells were transfected with 0.5 µg of pCMV-3xFLAG-ERα ABC, pCMV-3xFLAG-ERα ABCD, or pCMV-3xFLAG-ERα CDEF, and 1 µg of pEGFP-PARP7. The following day, cells were harvested and lysed in lysis buffer (200 mM NaCl, 20 mM Hepes, 1% Nonidet P-40). 10% of the lysate was kept as an input control, and the remaining lysate was incubated with 2 µg of anti-FLAG (Sigma-Aldrich; M2) and 20 µL of Dynabeads^TM^ Protein G (Thermo Fisher Scientific) with constant rotation for 2 h at 4 °C. The beads were washed five times with wash buffer (200 mM NaCl, 20 mM Hepes, 0.1% Nonidet P-40) and finally eluted in 2× Laemmli sample buffer supplemented with 10% β-mercaptoethanol. Samples were separated by SDS-PAGE and transferred to PVDF membranes. Membranes were incubated with anti-poly/mono-ADP-ribose (Cell Signaling Technology, Danvers, MA, USA; E6F6A), anti-FLAG (Sigma-Aldrich; F7425), and anti-GFP (Clontech Laboratories; JL-8). The 10% totals were incubated with anti-FLAG, anti-GFP and anti-β-actin. After incubation with appropriate secondary antibody, bands were visualized with SuperSignal^TM^ West Dura Extended Duration Substrate (Thermo Fisher Scientific). Quantification of modified FLAG-ERα was done in ImageLab^TM^ (BioRad) by normalizing to β-actin of the respective samples. 

### 2.12. Mass Spectrometry

GST-PARP7 was expressed and purified as previously described [17]. Ten micrograms of the protein were incubated with 0.5 mM NAD^+^ (Sigma-Aldrich), 1.5 µL of 20× ribosylation buffer (Trevigen, Gaithersburg, MD, USA), 1 µg of commercially available ERα (Thermo Fisher Scientific), and dH_2_O to a total volume of 30 µL. The reaction was incubated at room temperature for 30 min and stopped by adding 4× Laemmli sample buffer supplemented with 10% β-mercaptoethanol and boiled at 95 °C. Proteins were separated with SDS-PAGE, and the in-gel protein digestion, reverse phase nano liquid chromatography tandem mass spectrometry (LC-MS) analysis of proteolytic peptides, selection of LC-MS parameters and analysis was done as previously described [13]. 

### 2.13. Statistics

Data are presented as the standard error of the mean (S.E.M) of three individual replicates and analyzed with GraphPad Prism v8.2 (San Diego, CA, USA). Statistical analysis was carried out in the software using two-tailed student’s *t*-test or one-way analysis of variance (ANOVA) followed by Tukey’s post-hoc statistical test in order to correct for multiple comparisons where necessary. 

## 3. Results

### 3.1. PARP7 Expression is Induced by ERα

**Figure 1:**
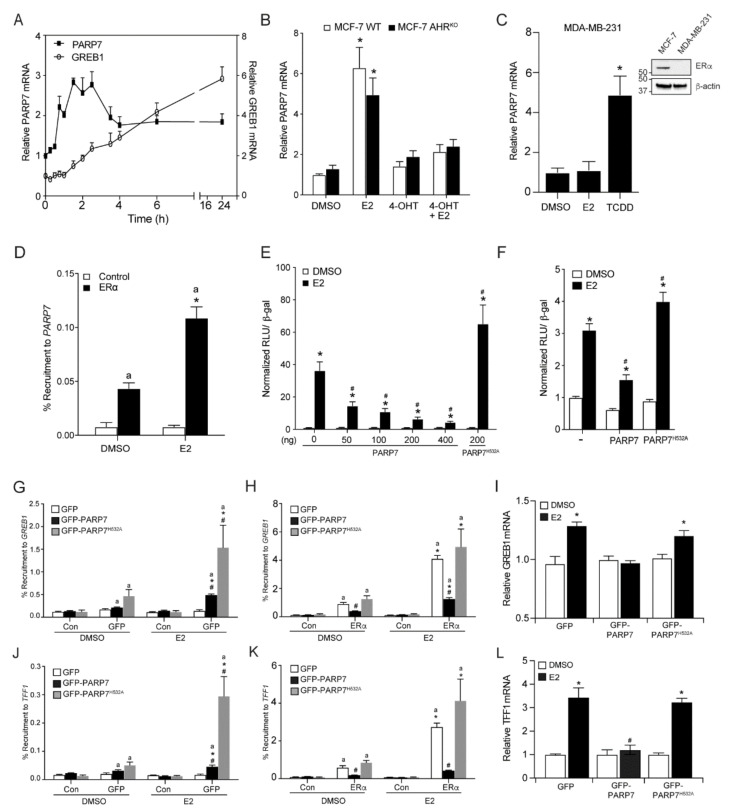
PARP7 is a target gene and repressor of ERα (**A**) PARP7 expression is induced by E2. MCF-7 cells were treated with E2, and RNA was isolated at various time points ranging from 15 min to 24 h. The relative mRNA levels of PARP7 (left axis) and GREB1 (right axis) were determined with RT-qPCR. (**B**) PARP7 is an ERα target gene and its expression is regulated by ERα, independent of AHR. MCF-7 wildtype and AHR^KO^ cells were treated with 0.1% DMSO, 10 nM E2 or/and 100 nM 4-OHT for 2 h. The co-treated samples were treated with 4-OHT 2 h prior to E2 treatment. The relative mRNA levels were determined with RT-qPCR. The asterisk * denotes significant differences (*p* < 0.05) from DMSO. (**C**) PARP7 mRNA levels in MDA-MB-231 cells treated with 0.1% DMSO, 10 nM E2 or 10 nM TCDD for 2 h. Insert western blot of ERα levels in MCF-7 compared with MDA-MB-231 cells. The asterisk * denotes significant differences (*p* < 0.05) from DMSO. (**D**) ERα is recruited to the PARP7 promoter in an E2-dependent manner. Wildtype MCF-7 cells were treated with 0.1% DMSO or 10 nM E2 for one hour. The letter “a” denotes recruitment differences significantly greater than the control (*p* < 0.05), and significant differences (*p* < 0.05) from DMSO is denoted with the asterisk *. PARP7, but not the catalytically inactive H532A mutant, repressed ERα-regulated reporter gene activity. (**E**) HuH-7 or (**F**) MCF-7 cells were transfected with ERE-TK-Luc reporter, pSG5-ERα, β-galactosidase, PARP7, or the catalytically inactive mutant. Six hours after transfection, cells were treated with 0.1% DMSO or 10 nM E2 for 18 h. Changes in reporter gene activity are shown as normalized relative light units (RLU). The asterisk * denotes significant differences (*p* < 0.05) from DMSO. Hash mark # denotes significant differences (*p* < 0.05) when compared to the response to E2 treatment in the absence of PARP7. (**G**) Overexpressed GFP-PARP7, and GFP-PARP7^H532A^ are recruited to *GREB1* in response to E2. GFP-PARP7 but not GFP-PARP7^H532A^ decreased (**H**) ERα binding to *GREB1* and (**I**) reduced GREB1 mRNA levels in transfected HuH-7 cells. Overexpressed (**J**) GFP-PARP7 and GFP-PARP7^H532A^ are recruited *TFF1* in response to E2. GFP-PARP7 but not GFP-PARP7^H532A^ decreased (**K**) ERα binding to *TFF1* and reduced (**L**) TFF1 mRNA levels in transfected HuH-7 cells. For G, H, J and K, recruitment differences significantly greater than the control (*p* < 0.05) are denoted with the letter “a”. Significant differences greater than antibody matched DMSO (*p* < 0.05) are denoted with the asterisk *. Significant differences greater than treatment matched GFP (*p* < 0.05) are denoted with the hash mark #. For I and L, the asterisk * denotes significant differences (*p* < 0.05) from transfection matched DMSO. Significant differences greater than transfection matched E2 (*p* < 0.05) are denoted with the hash mark #. Data are shown as means ± S.E.M. from three independent experiments.

To determine if PARP7 expression is regulated by E2, we treated MCF-7 cells with 10 nM E2 and prepared extracts at various time points from 15 min to 24 h and compared the mRNA levels of PARP7 to that of the E2-responsive gene, GREB1 (Figure 1A). This time course analysis revealed that PARP7 mRNA was induced by E2 treatment, but exhibited distinct temporal regulations compared with that of GREB1 (Figure 1A). The maximum PARP7 mRNA levels were observed between 1.5 and 2.5 h, whereas GREB1 mRNA levels reached a maximum at 24 h. We then determined if the E2-mediated regulation of PARP7 could be prevented by pharmacological inhibition of ERα and whether this regulation was independent of AHR, a well-known and potent regulator of PARP7 mRNA levels. We treated MCF-7 cells and MCF-7 AHR^KO^ cells with E2 in the presence or absence the ERα antagonist, 4-hydroxytamoxifen (4-OHT), for 2 h. The relative levels of PARP7 mRNA were determined by RT-qPCR. E2-treatment alone resulted in a significant increase in PARP7 mRNA levels in both cell lines (Figure 1B). Treatment with 4-OHT alone did not increase PARP7 expression, but prevented the ability of E2 to induce PARP7 mRNA levels. Treatment with E2, but not the AHR agonist, 2,3,7,8-tetrachlorodibenzo-*p*-dioxin (TCDD), failed to induce PARP7 mRNA levels in ERα negative MDA-MB-231 cells (Figure 1C). ChIP assays confirmed ERα recruitment to the *PARP7* promoter in an E2-dependent manner (Figure 1D). Taken together these data show that ERα regulated PARP7 expression in response to E2 independently of AHR. 

### 3.2. PARP7 Represses ERα Activity

We have previously reported that PARP7 acts as a negative regulator of AHR activity via mono-ADP-ribosylation [31]. To determine whether PARP7 represses ERα activity, we transfected HuH-7 cells with various amounts of PARP7, or the catalytically inactive mutant (PARP7^H532A^), together with ERα and an ERα-regulated luciferase reporter plasmid. Increasing amounts of PARP7 resulted in a dose- and ligand-dependent repression of ERα-regulated reporter gene activity (Figure 1E). Transfection of PARP7^H532A^ did not repress reporter gene activity, indicating that PARP7 must be catalytically active in order to repress ERα signaling. Transfection of PARP7^H532A^ also resulted in increased reporter gene activity compared with no PARP7. Similar findings were observed in transiently transfected MCF-7 cells (Figure 1F). In order to further understand how PARP7 affects ERα transactivation, ChIP assays were carried out in HuH-7 cells transfected with GFP-PARP7, or GFP-PARP7^H532A^, and ERα. E2 induced recruitment of GFP-PARP7 and GFP-PARP7^H532A^ to the regulatory region of *GREB1*. The recruitment levels of GFP-PARP7^H532A^ were significantly greater than those of GFP-PARP7 (Figure 1G). Decreased ERα binding and reduced GREB1 mRNA levels were observed in the presence of GFP-PARP7 but not GFP-PARP7^H532A^ (Figure 1H,I). Similar findings were observed for TFF1 (Figure 1J–L). These data provide evidence that catalytically active PARP7 negatively regulated ERα transactivation. 

### 3.3. The PARP7 Inhibitor, RBN-2397, Increases E2-Dependent GREB1 mRNA Levels and Stabilizes PARP7 and ERα Proteins

**Figure 2:**
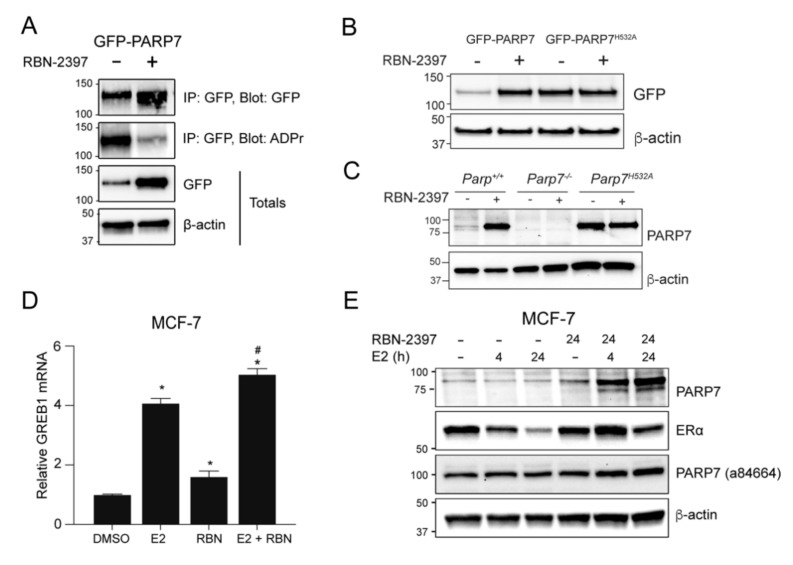
Inhibition of PARP7 activity stabilizes PARP7 protein levels and increases ERα activity. (**A**) RBN-2397 stabilizes PARP7 protein levels and decreases catalytic activity. COS-1 cells were transfected with GFP-PARP7 and treated with 0.1% DMSO or 100 nM RBN-2397 for 24 h. Samples were immunoprecipitated with anti-GFP, and membranes were blotted with anti-GFP and anti-ADP-ribose antibodies. (**B**) COS-1 cells were transfected with GFP-PARP7 or GFP-PARP7^H532A^ and treated with 0.1% DMSO or 100 nM RBN-2397 for 24 h. (**C**) *Parp7^+/+^*, *Parp7^−/−^* or *Parp7^H532A^* MEFs were treated with 0.1% DMSO or 100 nM RBN-2397 for 24 h. The membrane was probed with our lab generated anti-PARP7. (**D**) Treatment with RBN-2397 increases mRNA expression of ERα target gene GREB1. Wildtype MCF-7 cells were treated with 0.1% DMSO, 10 nM E2 or co-treated with E2 and 100 nM RBN-2397 for 24 h. The asterisk * denotes significant differences (*p* < 0.05) from DMSO, and the hash mark # denotes significant differences (*p* < 0.05) compared to E2 treatment alone. (**E**) E2 stimulation increases PARP7 protein expression. MCF-7 cells were treated with 10 nM E2 for 0, 4 and 24 h, together with control (no treatment) or 24 h treatment with 100 nM RBN-2397. The membrane was blotted with our lab generated anti-PARP7, anti-ERα, or anti-PARP7 (Abcam; ab84664) antibodies. PARP7 bands are visible in samples co-treated with E2 and RBN-2397. Anti-PARP7 (ab84664), did not detect endogenous PARP7, but rather detected a protein at approximately 100 kDa in all lanes.

Since we had observed the ability of PARP7 to inhibit ERα activity (Figure 1E,F), we investigated the effect of the small molecule PARP7 inhibitor, RBN-2397, on the PARP7-dependent regulation of ERα. ADP-ribosylation assays done on cell extracts isolated from COS-1 cells transfected with GFP-PARP7 and treated for 24 h with RBN-2397 confirmed RBN-2397′s ability to inhibit PARP7 catalytic activity (Figure 2A). In agreement with our previous data showing that the introduction of the point mutation H532A destroys PARP7 catalytic activity but also stabilizes PARP7 protein levels [17], treatment with RBN-2397 stabilized transfected GFP-PARP7 protein levels (Figure 2A,B). However, RBN-2397 did not affect the protein levels of GFP-PARP7^H532A^ (Figure 2B). We next determined the effect of RBN-2397 on the levels of endogenous PARP7 levels in *Parp7^+/+^*, *Parp7^−/−^* and *Parp7^H532A^* MEFs. Since we have been unable to identify a reliable commercially available anti-PARP7 antibody that detects endogenous protein, we generated a mouse monoclonal antibody against murine Parp7. Treatment of MEFs confirmed that RBN-2397 stabilizes endogenous Parp7 but does not affect the protein levels of Parp7^H532A^ (Figure 2C). In support of these data, treatment with RBN-2397 also stabilized endogenous PARP7 in E0771 murine triple negative breast cancer cells. However, due to a lack of ERα expression, co-treatment with E2 had no effect (Supplementary Figure S1A). Treatment of MCF-7 cells with E2 resulted in a significant increase in GREB1 mRNA levels. RBN-2397 treatment alone also significantly increased GREB1 mRNA levels compared with DMSO, but to a significantly lower level than those induced by E2 (Figure 2D). Co-treatment of E2+RBN-2397 resulted in a slight, but significantly higher increase in GREB1 mRNA levels compared with E2 alone (Figure 2D). 

To determine whether E2 treatment also results in increased endogenous PARP7 protein levels, MCF-7 cells were treated with 10 nM of E2 for 4 and 24 h. Transfection with different N-terminal truncations of GFP-PARP7 (human) revealed that our anti-PARP7 antibody recognizes a region within the N-terminus in PARP7 that includes 1–53 a.a. (Supplementary Figure S1B,C). A comparison between our lab-generated antibody and a commonly used commercially available anti-PARP7 antibody confirmed its increased selectivity for PARP7 (Supplementary Figure S1B,C). Our lab generated antibody was raised against murine Parp7, but it also cross-reacts with human PARP7, albeit with reduced sensitivity. In E2 treated MCF-7 cells, we were unable to detect increased PARP7 protein levels at either timepoint (Figure 2E). This was most likely due to rapid turnover or instability of PARP7 [32] and a low sensitivity of anti-PARP7 antibody to detect human PARP7. However, PARP7 protein levels were increased in cells co-treated with E2+RBN-2397 for 4 or 24 h compared with RBN-2397 alone. This indicated that PARP7 protein expression is induced by E2, but that the inhibition PARP7 catalytic activity was necessary to stabilize PARP7 protein levels to detect the protein with our antibody. The detected band was slightly higher than PARP7′s predicted 76 kDa molecular weight, but similar to that observed in MEFs (Figure 2C and Supplementary Figure S1C). The findings, however, support previous studies of transfected full length and truncated PARP7 that show that it runs higher than its predicted weight [17]. A commercially available anti-PARP7 (a84664) failed to detect PARP7 after co-treatment with E2 and RBN-2397. A strong band at approximately 100 kDa was detected in all lanes. Interestingly, E2-dependent decreases in ERα protein levels were reduced upon PARP7 inhibition, suggesting that PARP7 regulates ERα proteolytic degradation. 

### 3.4. Generation of CRISPR/Cas9-Mediated PARP7 Knockout MCF-7 Cells

**Figure 3:**
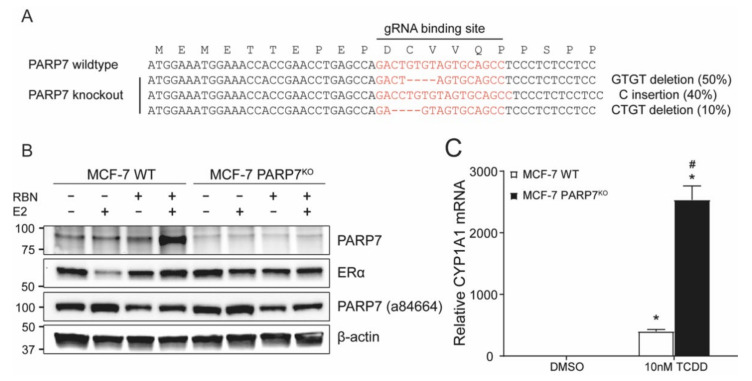
Confirmation of MCF-7 PARP7 knockout cells. (**A**) Schematic representation of the gRNA binding site, showing insertions/deletions resulting in frameshift mutations. The deleted bases are represented as dashes. The data are from 45 independent sequences. (**B**) PARP7 is not detected in the PARP7^KO^ cells when blotting the membrane with our lab generated anti-PARP7 antibody. MCF-7 wildtype and PARP7^KO^ cells were treated with DMSO, E2 or/and RBN-2397 for 24 h. Membranes were blotted with lab generated anti-PARP7, anti ERα and anti-PARP7 (Abcam; ab84664). Blotting with anti-PARP (ab84664) resulted in bands across all lanes at approximately 100 kDa. (**C**) Loss of PARP7 increases expression of AHR target gene CYP1A1. MCF-7 wildtype and PARP7^KO^ cells were treated with 10 nM TCDD for 24 h. Relative mRNA levels of CYP1A1 was determined with RT-qPCR. Significant differences (*p* < 0.05) compared to DMSO are denoted with *, and differences due to PARP7 are denoted with the hash mark #. Data are shown as means ± S.E.M. for three independent experiments.

To further study the interplay between PARP7 and ERα, we generated CRISPR/Cas9-mediated PARP7 knockout (PARP7^KO^) MCF-7 cells. Sequencing of a portion of the PARP7 gene surrounding the gRNA binding site after puromycin selection identified insertions/deletions resulting in frameshift mutations in PARP7 (Figure 3A). To confirm PARP7 knockout, MCF-7 wildtype and PARP7^KO^ cells were treated with E2 or/and RBN-2397 in order to induce expression of, and stabilize PARP7. When probed with our lab-generated anti-PARP7, there were no visible bands in the PARP7^KO^ samples (Figure 3B). However, when probing the membrane with anti-PARP7 (ab84664), we observed a band at 100 kDa in all lanes. In line with previous observations, E2-dependent decreases in ERα protein levels were reduced in the PARP7^KO^ cells. To provide further verification of PARP7 knockout, MCF-7 wildtype and PARP7^KO^ cells were treated with TCDD for 24 h, a potent AHR ligand, and the relative mRNA levels of the AHR target gene CYP1A1 were determined. CYP1A1 mRNA was significantly higher in the knockout cells, indicating that the repressive role of PARP7 on AHR activity was abolished (Figure 3C). 

### 3.5. PARP7 Knockout MCF-7 Cells Display Increased ERα Activity and E2-Induced Proliferation

**Figure 4:**
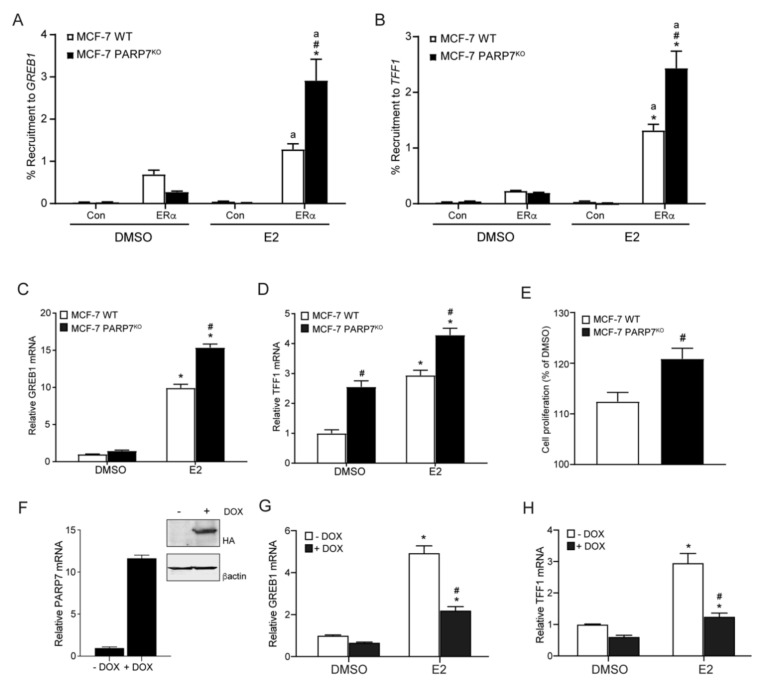
MCF-7 PARP7^KO^ cells display increased ERα activity. Recruitment of ERα to the regulatory regions of (**A**) *GREB1* and (**B**) *TFF1* was increased in MCF-7 PARP7^KO^ cells compared with MCF-7 wildtype cells. Cells were treated with 0.1% DMSO or 10 nM E2 for one hour. Chromatin immunoprecipitation was carried out with no antibody (control) or anti-ERα. Recruitment differences significantly greater than the control (*p* < 0.05) are denoted with the letter “a”, differences due to PARP7 (*p* < 0.05) are denoted with the hash mark #. Significant differences greater than DMSO (*p* < 0.05) are denoted with the asterisk *. (**C**) GREB1 and (**D**) TFF1 mRNA levels were significantly higher in MCF-7 PARP7^KO^ cells treated with 10 nM E2 for 24 h compared with MCF-7 wildtype cells. The asterisk * denotes statistical significance (*p* < 0.05) when compared to the DMSO-treated wildtype, and hash mark # denotes significant (*p* < 0.05) differences due to PARP7. (**E**) MCF-7 PARP7^KO^ cells exhibited increased proliferation in response to E2 compared with wildtype cells. Cells were treated with 0.1% DMSO or 10 nM E2 every day for 4 days. Cell proliferation was normalized to baseline (Day 1) and to the DMSO-treated samples. The hash mark # denotes statistical significance (*p* < 0.05) between the cell lines. (**F**) MCF7 cells expressing a Tet-ON regulated HA-tagged PARP7 were cultured in the presence or absence of doxycycline (DOX) to induce (**F**) PARP7 mRNA and protein levels (inset F). Cells were then incubated with or without DOX and treated with DMSO or 10 nM of E2 for 20 h. The mRNA expression levels of (**G**) GREB1 and (**H**) TFF1 were reduced in the presence of DOX. The data are representative of three independent experiments. The asterisk * denotes significant difference (*p* < 0.05) greater than DMSO, and the hash mark # denotes differences due to DOX. Data are shown as means ± SEM for three independent experiments.

We next determined the E2-dependent ERα recruitment to GREB1 and TFF1 in MCF-7 wildtype and PARP7^KO^ cells. Cells were treated with DMSO or E2 for one hour prior to doing ChIP qPCR assays. We observed increased E2-induced recruitment of ERα to the promoter region of *GREB1* (Figure 4A) and *TFF1* (Figure 4B) in MCF-7 PARP7^KO^ compared with wildtype cells. Cells were then treated with DMSO or E2 for 24 h and the relative amounts of GREB1 and TFF1 mRNA levels were determined. Significantly higher E2-dependent increases GREB1 (Figure 4C) and TFF1 (Figure 4D) mRNA levels were observed in MCF-7 PARP7^KO^ compared with wildtype cells. Interestingly, mRNA levels of TFF1 in the DMSO-treated MCF-7 PARP7^KO^ cells were significantly higher than those of MCF-7 wildtype cells. This could indicate that PARP7 also negatively regulates ERα in the absence of ligand. 

Since E2 supports proliferation of ER positive breast cancer cells [33], we hypothesized that MCF-7 PARP7^KO^ cells would exhibit increased proliferation in response to E2. To test this, the proliferation of MCF-7 wildtype and PARP7^KO^ cells was measured after being exposed to E2 for 4 days (Figure 4E). Consistent with our hypothesis, our data showed significantly increased proliferation in the MCF-7 PARP7^KO^ cells compared to wildtype cells, indicating that endogenous PARP7 negatively affects breast cancer cell proliferation, possibly due to repression of ERα. To determine the effect of PARP7 expression on endogenous ERα signalling, we used a Tet-ON PARP7 overexpression cell line, in which the expression of HA-tagged PARP7 is regulated by doxycycline (DOX). Treatment with 1.5 μg/mL DOX resulted in an approximate 12-fold increase in PARP7 mRNA levels (Figure 4F) and increase in PARP7-HA protein levels (inset Figure 4F). E2-induced GREB1 and TFF1 mRNA levels were reduced in the presence of DOX compared with E2 treatment alone (Figure 4G,H). These results show that overexpression of PARP7 negatively regulates endogenous ERα activity. 

### 3.6. Overexpressed PARP7 Mono-ADP-ribosylates Overexpressed ERα

**Figure 5:**
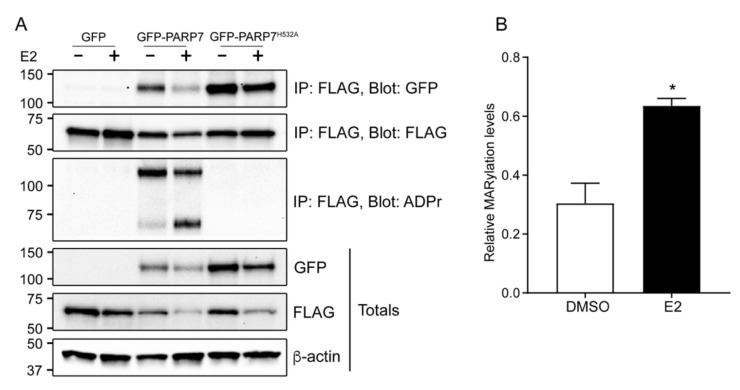
Overexpressed PARP7 mono-ADP-ribosylates overexpressed ERα. (**A**) PARP7 and its catalytically inactive mutant interacts with ERα in COS-1 cells when treated with DMSO and E2. COS-1 cells were transfected with FLAG-ERα, and either GFP, GFP-PARP7 or GFP-PARP7^H532A^, and treated with DMSO or E2 for 24 h. Co-immunoprecipitation was carried out with anti-FLAG. The membranes were incubated with anti-FLAG, anti-GFP, and anti-ADP-ribose. Both wildtype PARP7 and ERα are mono-ADP-ribosylated, but not by the catalytically inactive PARP7^H532A^ mutant. (**B**) Relative MARylation levels of immunoprecipitated FLAG-ERα in the presence of GFP-PARP7 after treatment with DMSO or E2 for 24 h. Quantification of protein bands normalized to β-actin revealed that mono-ADP-ribosylation of ERα was significantly (*p* < 0.05) increased upon treatment with E2 as indicated by an asterisk *.

To investigate the interaction between PARP7 and ERα, we performed co-immunoprecipitation assays. COS-1 cells were transfected with FLAG-ERα, and either GFP, GFP-PARP7 or GFP-PARP7^H532A^. The cells were treated with DMSO or E2 for 24 h. We observed that both overexpressed PARP7 and its catalytically inactive mutant co-immunoprecipitated with overexpressed ERα in all conditions, indicating that the interaction between the proteins is independent of PARP7′s catalytic activity (Figure 5A). Overexpressed PARP7 wildtype, but not its catalytically inactive mutant, mono-ADP-ribosylated overexpressed ERα (Figure 5A). Transfected ERα was mono-ADP-ribosylated in both the DMSO and E2 treated samples, but to a significantly higher extent in response to E2 (Figure 5B). Moreover, both overexpressed wildtype PARP7 and overexpressed ERα levels were decreased in response to E2. It is well established that E2 induces proteolytic degradation of ERα [34], yet this was more prominent in the samples co-expressing wildtype PARP7. These results showed that overexpressed PARP7 mono-ADP-ribosylated overexpressed ERα in response to E2, which led to decreased ERα protein levels. 

### 3.7. Identification of Mono-ADP-ribosylated Peptides in Bacterial Expressed and Purified ERα

**Figure 6:**
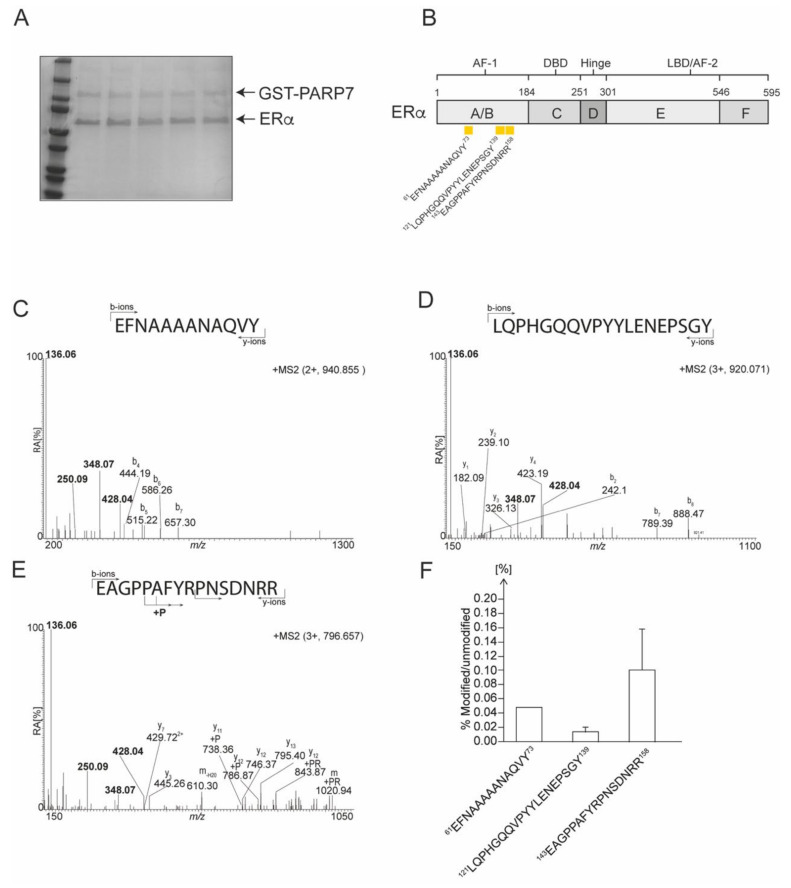
Identification of mono-ADP-ribosylated peptides in bacterial expressed and purified ERα. (**A**) Representative SDS-PAGE of GST-PARP7 and ERα prior to LC/MS analysis. (**B**) A schematic representation of the domain structure of ERα. Location of ADP-ribosylated peptides are denoted by yellow rectangles. Peptide sequences are numbered from the unmodified full-length protein. (**C**) The MS2 spectrum of the trypsin generated ion at *m/z* 940.855. (**D**) The MS2 spectrum of the trypsin generated ion at *m/z* 920.705. (**E**) The MS2 spectrum of the trypsin generated ion at *m/z* 796.657. (**F**) Relative levels of modification (in percentage) of ADP-ribosylated peptides identified by M/S in ERα.

To determine the location of the ADP-ribosylated peptides in ERα, we analyzed the ability of bacterial expressed GST-PARP7 to ADP-ribosylate purified ERα *in vitro*. After co- incubating the proteins in the presence of NAD^+^, the proteins were separated by SDS-PAGE (Figure 6A) and the respective bands excised prior to performed LC/MS analyses. We used both trypsin- and chymotrypsin-digestion to identify in vitro ADP-ribosylated ERα peptides by mass spectrometry utilizing HCD fragmentation and investigated the generated MS2 peptide spectra for the presence of the specific ADP-ribose reporter ions at *m/z* 250.09, at *m/z* 348.07 and at *m/z* 428.04 (Supplementary Figures S2 and S3). Peptide spectra displaying all three reporter ions were considered ADP-ribosylated, and we considered peptide forms with sequence overlap due to missed enzyme cleavage sites, sodium adducts, and oxidized forms as a single unique ADP-ribosylated peptide. We identified three unique in vitro mono-ADP-ribosylated peptides in ERα (Figure 6B). All of the identified in vitro mono-ADP-ribosylated peptides eluted as a single peak in the chromatogram, and none of the modified peptides were detected with more than a single mono-ADP-ribosylation, indicating that only a single site was modified on each mono-ADP-ribosylated peptide. The doubly charged precursor ion at *m/z* 940.855 (observed mass of 1880.703 Da [M+H+]) corresponded to the peptide ^61^EFNAAAAANAQVY^73^ (theoretical mass 1339.628 [M+H+]) carrying a single ADP-ribose (theoretical mass of 541.061 Da). All three ADP-ribose specific reporter ions were detected in the low mass area of the MS2 spectrum (Figure 6C). The b-ions b_7_-H_2_O (at *m/z* 657.30), b_6_-H_2_O (at *m/z* 586.26), b_5_-H_2_O (at *m/z* 515.22), b_4_-H_2_O (at *m/z* 444.19), as well as the y_1_-ion (at *m/z* 182.08) were identified in the MS2 spectrum, verifying the amino acid sequence of the peptide. The triply charged peptide at *m/z* 920.705 (observed mass of 2758.198 Da [M+H+]) corresponded to the peptide ^121^LQPHGQQVPYYLENEPSGY^139^ (theoretical mass 2219.040 Da [M+H+]) carrying a single ADP-ribose (Figure 6D). All three ADP-ribose specific reporter ions were detected in the low mass area of the MS2 spectrum. A number of b- and y-ions were identified in the MS2 spectrum, verifying the amino acid sequence of the peptide. The triply charged precursor ion at *m/z* 796.657 corresponded to the peptide ^143^EAGPPAFYRPNSDNRR^158^ (theoretical mass 1846.894 Da [M+H+]) carrying a single ADP-ribose (Figure 6E). All three ADP-ribose specific reporter ions were detected in the low mass area of the MS2 spectrum. A number of b- and y-ions was identified in the MS2 spectrum, verifying the amino acid sequence of the peptide. Interestingly, a series of y-ions were detected carrying a mass adjustment corresponding to a phosphate addition (79.97 Da) or phospho-ribose addition (193.97 Da) starting at y11 (A148), indicating that the ADP-ribose is located within the sequence ^148^AFYRPNSDNRR^158^. Interestingly a phosphate addition was also detected on the y-ion series indicating perhaps that PARP7 can connect ADP-ribose to the peptide backbone or that ADP-ribose can undergo rearrangements during HCD analysis in a similar fashion as seen for glycans [35]. 

All three peptides were located in the N-terminal A/B domains of ERα. To quantify the relative abundance of modified versus unmodified peptide we utilized the AUC (area under the curve) approach and considered the total peptide population that represented all peptides formed, including peptide forms with sequence overlap due to missed trypsin cleavage sites, sodium adducts, and oxidized forms. Although the overall level of mono-ADP-ribosylation was low on all peptides, the ^143^EAGPPAFYRPNSDNRR^158^ peptide displayed the highest level of modification at an average of 0.11% relative to the unmodified form, with the ^61^EFNAAAAANAQVY^73^ and ^121^LQPHGQQVPYYLENEPSGY^139^ peptides displaying a relative level of modification at 0.05% and 0.01%, respectively (Figure 6F). However, the level of modification varied greatly between samples with as much as a ten-fold difference for the modified ^143^EAGPPAFYRPNSDNRR^158^ peptide. To identify the ADP-ribosylated amino acid residues in the respective peptides, electron transfer dissociation (ETD) fragmentation was done as we have previously described [13]. However, these studies were unsuccessful and as a result, we were unable to identify the specific ADP-ribose acceptor residues in the peptides. Overall, these data show that in vitro mono-ADP-ribosylation occurred on at least three different peptide sequences in ERα, all of which were located within the AF-1 domain.

### 3.8. The Hinge Region of ERα is Required for its Mono-ADP-ribosylation by PARP7

**Figure 7:**
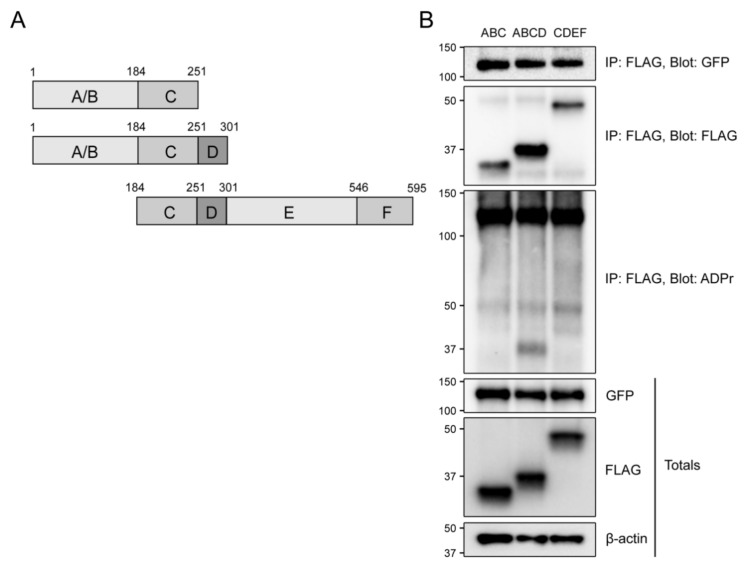
The hinge region of ERα is required for mono-ADP-ribosylation by PARP7. (**A**) A schematic representation of the truncated variants of ERα. (**B)** PARP7 co-immunoprecipitated with all three ERα variants. Only ERα ABCD was mono-ADP-ribosylated. COS-1 cells were transfected with GFP-PARP7 and either 3xFLAG-ERα ABC, 3xFLAG-ERα ABCD, 3xFLAG-ERα CDEF. Co-immunoprecipitation was carried out with anti-FLAG, and membranes were blotted with anti-FLAG, anti-GFP and anti-ADP-ribose.

In order to confirm the mono-ADP-ribosylation of ERα’s transactivating AF-1 domain, we performed a co-immunoprecipitation assay with three truncated variants of ERα (Figure 7A). COS-1 cells were transfected with GFP-PARP7 and FLAG-ERα variants ABC, ABCD, and CDEF. Since only CDEF contained a ligand binding domain, the samples were not treated with E2. As expected, we did not observe any mono-ADP-ribosylation in ERα CDEF, which lacked the A/B domain (Figure 7B). Surprisingly, no mono-ADP-ribosylation was detected in the ABC variant, although it contained the AF-1 domain. We did, however, detect mono-ADP-ribosylation of the ABCD variant, with an intensity comparable to the DMSO-treated sample in Figure 5A. This could imply that the D domain, or hinge region, is required for the modification to occur on ERα. 

## 4. Discussion

Here we provide evidence that PARP7 is part of a negative feedback loop regulating ERα activity. Inhibition of PARP7 resulted in increased ERα protein levels and signaling, while overexpression of PARP7 repressed ERα activity and decreased its recruitment to its target gene promoters. In support of these data, PARP7 knockout cells displayed increased ERα activity, as shown by increased mRNA expression levels of and recruitment of ERα to target genes, and increased cell proliferation in response to E2. ERα was mono-ADP-ribosylated by PARP7 in response to E2, and PARP7′s ability to repress ERα was dependent on its catalytic activity. Taken together, these data illustrate the importance of PARP7 and mono-ADP-ribosylation in the regulation of ERα activity, and possibly cell proliferation in ER positive breast cancers.

Inhibiting PARP7 catalytic activity stabilized its protein levels but also those of ERα. PARP7 has also been reported to regulate AHR protein levels [17,31], and more recently PARP7 has been shown to recruit both HIF-1α and an E3 ubiquitin ligase HUWE1 to nuclear bodies to promote the ubiquitination and degradation of HIF-1α [19]. We provide evidence implicating mono-ADP-ribosylation of ERα as a mechanism to regulate its protein stability. However, the events leading to degradation of ERα and whether PARP7 recruits ERα together with an E3 ubiquitin ligase to nuclear bodies remain elusive. AHR functions as an E3 ligase to regulate ERα and other oncogenic transcription factor levels [36]. Given the importance of PARP7 in AHR signaling it is tantalizing to speculate that PARP7 functions in concert with AHR to regulate the protein levels of these and other oncogenic transcription factors. 

Due to the low levels of detected mono-ADP-ribosylated peptides, we were unable to identify target residues in ERα, but three mono-ADP-ribosylated peptides in ERα were mapped to the receptor’s ligand independent transactivation domain, AF-1. In vitro ADP-ribosylation assays failed to confirm the mono-ADP-ribosylation in AF-1 (AB domains) without the presence of the D domain. The truncated CDEF variant (AF-1 deficient) was not mono-ADP-ribosylated by PARP7, suggesting that the D domain is not mono-ADP-ribosylated. How the D domain influences the ability of PARP7 to modify AF-1 region of ERα is unknown. We cannot, however, exclude the possibility that there are additional mono-ADP-ribosylated residues in ERα that we were unable to identify using purified ERα and PARP7 proteins. Moreover, it is possible that mono-ADP-ribosylated peptides identified in heterologous expressed and purified ERα may not reflect peptides or amino acid residues that are mono-ADP-ribosylated in vivo. Recent studies using enrichment of ADP-ribosylated proteins by incubation with the macrodomain protein, AF1521, have revealed that ADP-ribosylation occurs on a number of distinct amino acids, including acidic residues (Glu/Asp), arginine (Arg), serine (Ser), tyrosine (Tyr), histidine (His), and cysteine (Cys) [37,38]. Many of these residues are present in the identified peptides and could represent potential ADP-ribose acceptor sites in ERα. The application of ADP enrichment strategies, which have been utilized to characterize the ADP-ribosylome, could be used to map mono-ADP-ribosylation sites in ERα. It is important to note, however, that the in vitro ADP-ribosylation studies done using purified proteins may not accurately reflect mono-ADP-ribosylation sites in PARP7 that occur in vivo. 

Based on the results presented in here, we hypothesize that PARP7 functions as a tumor suppressor in E2 responsive breast cancer cells by repressing the oncogenic actions of ERα. In support of this, a recent study reported that PARP7 knockdown promoted tumor growth in an MCF-7 xenograft model [19]. However, in contrast to this finding, the PARP7 inhibitor used in this study, RBN-2397, has been reported to cause cancer regression in xenograft mouse models [21]. PARP7 is a key regulator of innate immunity by repressing TBK1, an important regulator of IFN-I signaling [20]. By inhibiting PARP7, the IFN-I signaling axis is restored, enabling immune cells to target cancer cells. RBN-2397, which exploits PARP7′s role in regulating the IFN-I signaling, is currently in a Phase 1 clinical trial designed to assess its anti-tumor activity in patients with advanced-stage solid tumors (NCT04053673). Whether PARP7 inhibition-induced immune cell targeting of cancer cells overrides the increased activity of oncogenic transcription factors, such as ERα, remains to be determined. It would be interesting to compare the role of PARP7 in cancer cells versus host immune cells in syngeneic in vivo models.

The ADP-ribose glycohydrolase, MACROD1 (LRP16), has been reported to interact with the AF-1 domain of ERα, and enhance ERα transcriptional activity [39]. This report, and our findings, imply that PARP7 and MACROD1 might work in concert to regulate ERα activity by catalyzing the transfer or removal of ADP-ribose on the receptor’s AF-1 domain and may represent novel targets for ER positive breast cancer treatment. Recent studies have, however, shown that MACROD1 is almost exclusively expressed in the mitochondria [40]. Therefore, the possible role of MACROD1 as a co-regulator of transcription factor activity and its potential functions in the nucleus need to be fully clarified. It is possible that MACROD1 influences the cellular pool of NAD^+^ or additional mitochondrial processes that indirectly impact ERα function.

We only examined PARP7 function in ER positive breast cancer cells. Whether or not PARP7 has a tumor suppressive effect in other subtypes of breast cancer is not known. Previous studies have shown that the androgen receptor (AR) regulates PARP7 expression [41,42]. In addition, MACROD1 has been reported to act as a co-activator in AR signaling [43], inferring that AR could be a target for PARP7-mediated mono-ADP-ribosylation. Since AR has been proposed as an important regulator of carcinogenesis in a subset of triple negative breast cancers (TNBC), the interplay between PARP7 and AR could be studied to further understand the role of PARP7 in breast cancer. 

In summary, we show that PARP7 negatively regulates ERα, establishing a link between PARP7-mediated mono-ADP-ribosylation and ERα signaling. Further studies are needed to determine how mono-ADP-ribosylation affects ERα protein levels and stability. In addition to promoting the degradation of AHR, HIF1α and ERα, PARP7 also promotes the degradation of c-Myc [19], suggesting that PARP7 may represent a key regulatory factor controlling and suppressing the expression of several oncogenic transcription factors. In contrast, PARP7 is a negative regulator of IFN-I signaling, which allows tumor cells to “hide” from immunosurveillance. Thus, inhibition or loss of PARP7 expression would be expected to prevent tumors from evading the immune system, leading to increased anti-tumorigenic responses. It will be important to determine whether PARP7′s immunomodulatory role, which may induce immune cell mediated tumor cytotoxicity, supersedes the increased activity of oncogenic transcription factors.

## Data Availability

All data are included in the paper. There are no databases associated with this manuscript.

## Supplementary Materials

The following are available online at https://www.mdpi.com/2073-4409/10/3/623/s1, Figure S1: Characterization of lab generated anti-PARP7 antibody in COS1 cells and E0771 cells. Figure S2: Moment of precursor ion selection for MS2 fragmentation leading to ADP-R specific reporter ions in the generated fragment spectra. Figure S3: Liquid chromatography and mass spectrometry (LC/MS) of protein samples used for the identification of peptides in ERα.
