# Supplementary material for: PARP7 and Mono-ADP-Ribosylation Negatively Regulate Estrogen Receptor α Signaling in Human Breast Cancer Cells
Source: Cells. 2021 Mar 11;10(3):623. doi: 10.3390/cells10030623 (PMC8001409; doi:10.3390/cells10030623)
Supplement: Supplementary file 1 [file cells-10-00623-s001.pdf]

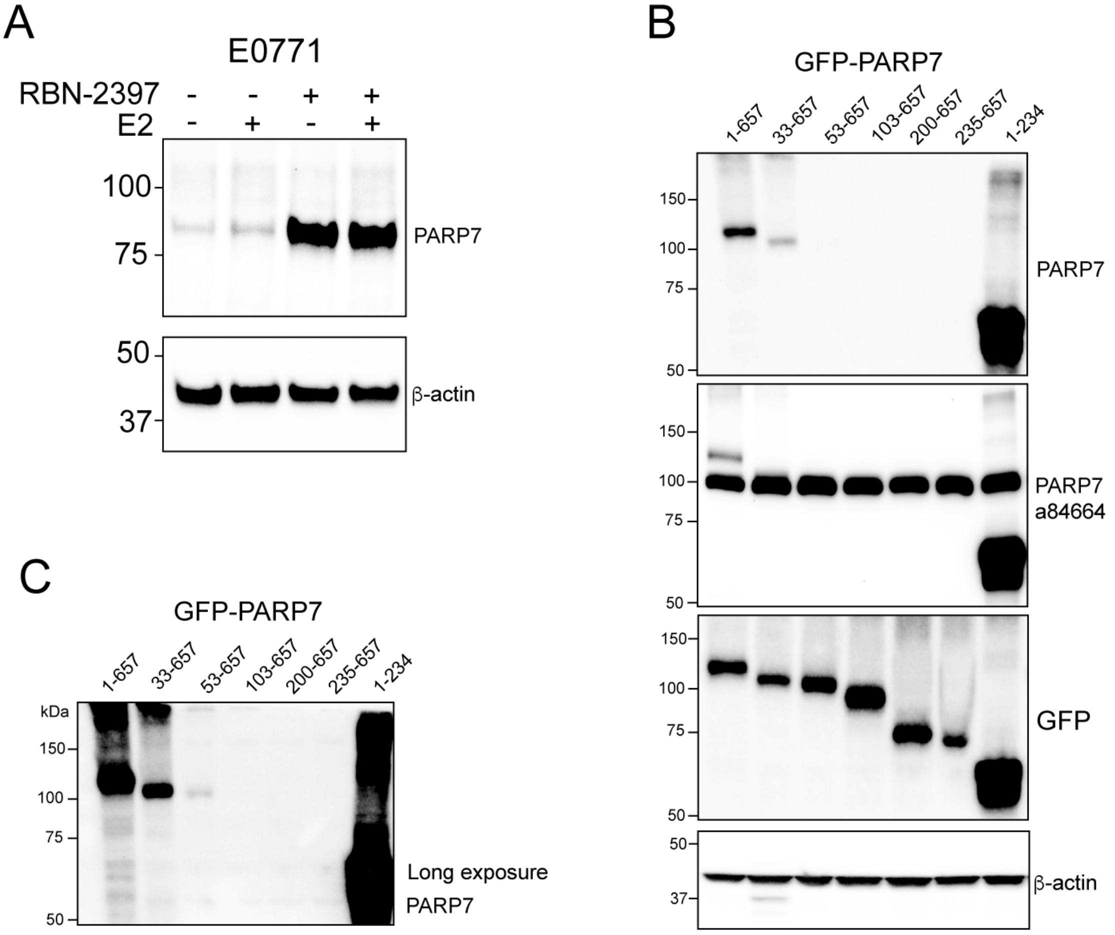

Supplementary Figure S1. Characterization of lab generated anti-PARP7 antibody. A) In-house generated anti-PARP7 antibody recognized endogenous murine Parp7. Mouse breast cancer, E0771, cells were treated with 0.1% DMSO, 10 nM E2, or/and 100 nM RBN-2397 for 24 h. Membrane was blotted with lab generated anti-PARP7 (1:1000) monoclonal antibody as described in Materials and Methods. **B)** In-house generated anti-PARP7 antibody recognizes an N-terminal portion of human PARP7, and has increased selectivity compared to anti-PARP7 (a84664). COS-1 cells were transfected with different N-terminal truncations of GFP-PARP7. The membrane was blotted with lab generated anti-PARP7 (1:1000), anti-PARP7 (Abcam; a84664, 1:1000) and anti-GFP (Clontech; JL8, 1:2000). **C)** Increased exposure of the membrane blotted with lab generated anti-PARP7 antibody revealed that it recognizes a region including a.a. 1-53 of PARP7.

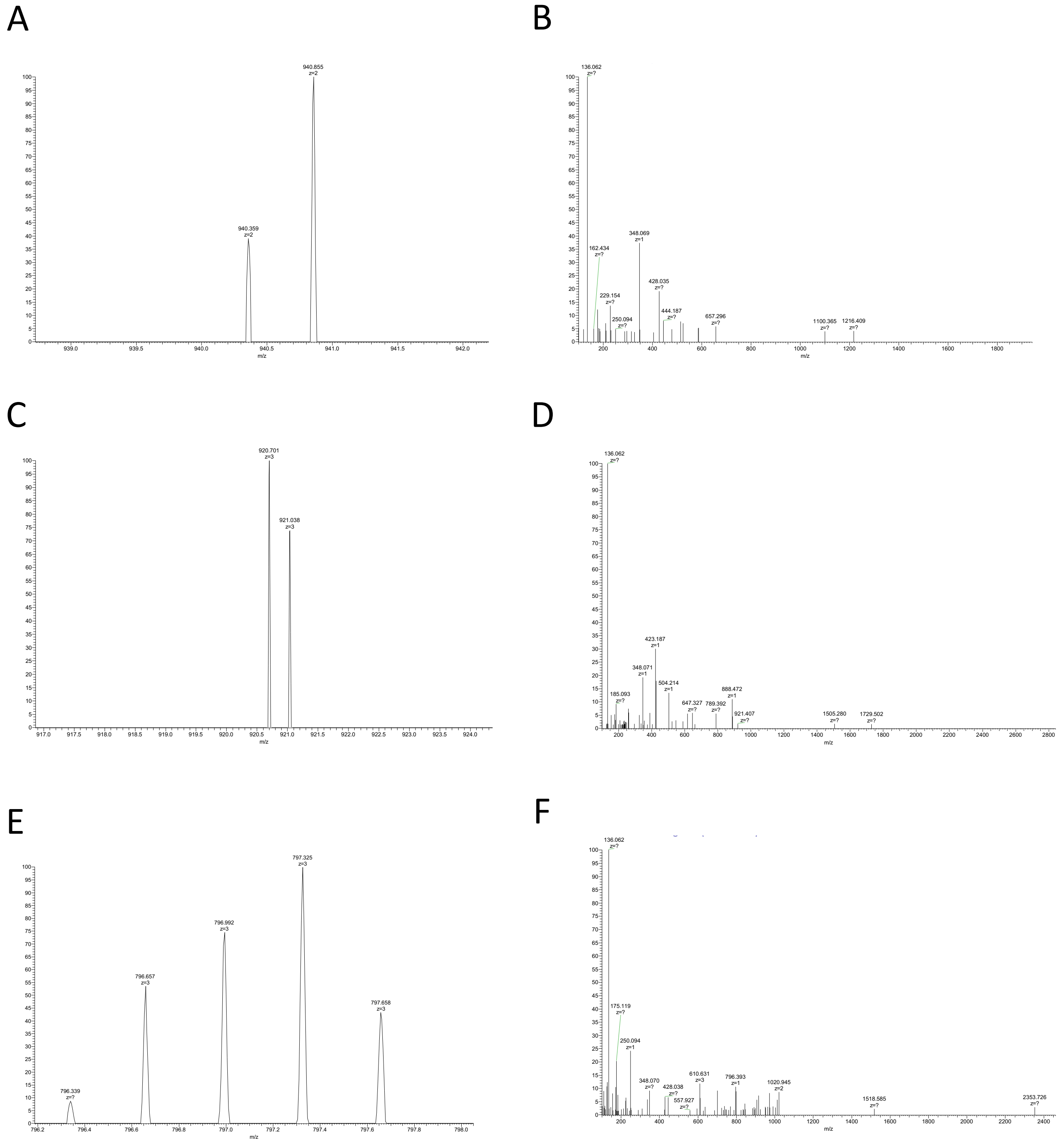

Supplementary Figure S2. Moment of precursor ion selection for MS2 fragmentation leading to ADP-R specific reporter ions in the generated fragment spectra. The absence of other molecules in the selection window indicate that only the selected molecules were fragmented and generated a combination of sequence specific ions and ADP-R ions in the MS2 spectra that were indicative of a modified peptides. A) the doubly charged precursor at m/z 940.855 yielding the B) fragment spectrum consistent with an ADP-R modified EFNAAAAANAQVY peptide when selected for fragmentation. C) the triply charged precursor at m/z 921.035 yielding the D) fragment spectrum consistent with a modified LQPHGQQVPYYLENEPSGY peptide when selected for fragmentation. E) the triply charged precursor at m/z 797.325 yielding the F) fragment spectrum consistent with an ADP-R modified EAGPPAFYRPNSDNRR peptide when selected for fragmentation.

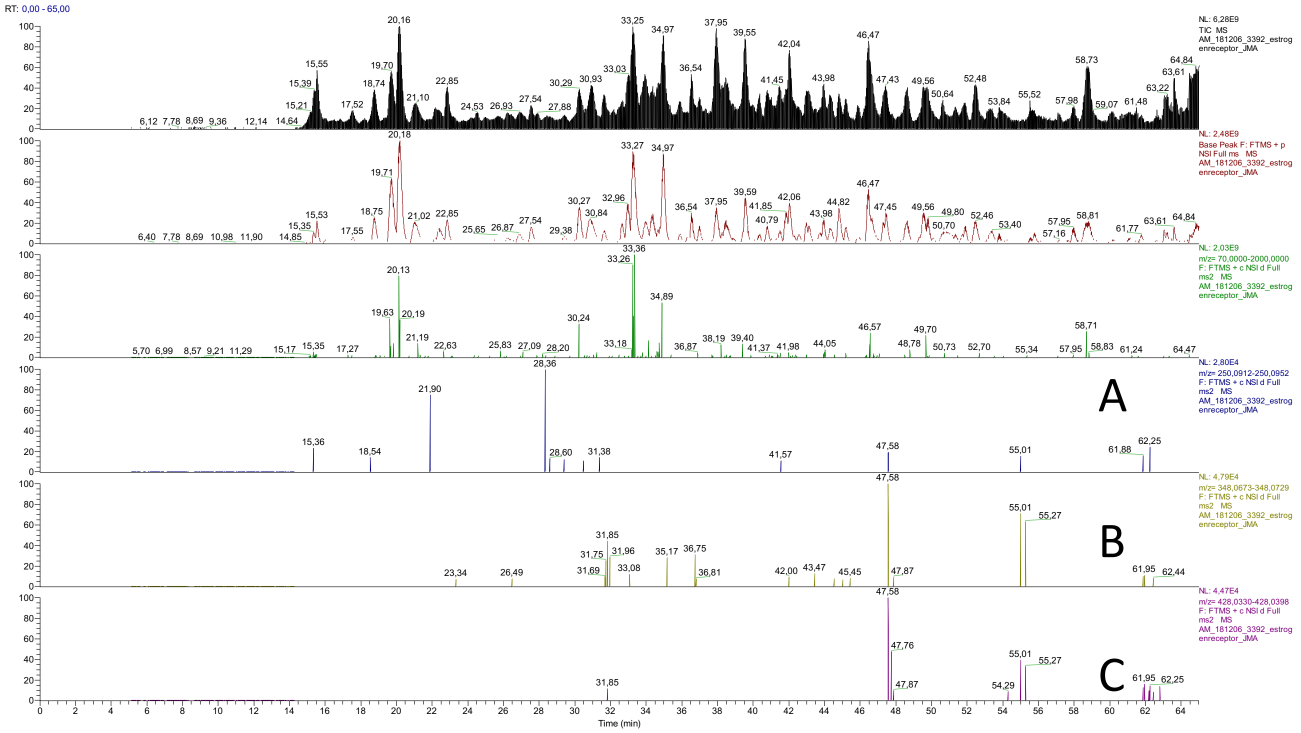

Supplementary Figure S3. Liquid chromatography and mass spectrometry (LC/MS) of protein samples used for the identification of peptides EFNAAAANAQVY and LQPHGQQVPYYLENEPSGY. The chromatograms marked A,B and C show peaks representing the three specific reporter ions for ADP-R . Only MS2 spectra with complete overlap of all three peaks were considered a ADP-R positive spectra, which were then further investigated manually. Very few positions in the chromatogram showed overlap of peaks from A (250.09) , B (348.07) and C (428.04) indicating that the presence of ADP-R was limited to only a few spectra.
